# The diagnostic value of confocal laser endomicroscopy in a cancer-mimicking lesion

**DOI:** 10.1055/a-2671-0168

**Published:** 2025-08-14

**Authors:** Yujie Shi, Qin Zeng, Yuxuan Chen, Mi Zhou, Shuo Zhang

**Affiliations:** 1 70571 Zhejiang Chinese Medical University, Hangzhou, China; 2 587400 Department of Gastroenterology, The Second Affiliated Hospital of Zhejiang Chinese Medical University, Hangzhou, China; 3 Key Laboratory of Traditional Chinese Medicine for the treatment of Intestine-Liver of Zhejiang Province, Hangzhou, China

A 25-year-old woman presented with epigastric pain for two months. Laboratory tests revealed mild anemia (hemoglobin: 114 g/L) and elevated C-reactive protein (8.9 mg/L). Treponema pallidum antibody and rapid plasma reagin (RPR) tests were positive (titer: 1:32), while tumor markers, fecal occult blood test, hepatitis B surface antigen, and HIV screening were all negative. Abdominal computed tomography showed only a left renal cyst. One month earlier, the patient had undergone gastroscopy at a local hospital, which revealed a large irregular ulcer at the gastric angle. Biopsy suggested chronic atrophic gastritis with erosion. Although immunohistochemistry excluded lymphoma, the lesion appeared highly suspicious, and the patient was referred to our center for further evaluation.

Repeat gastroscopy confirmed an irregular ulcer at the gastric angle, surrounded by nodular elevated mucosa ( Fig. 1 **a** ). After staining the lesion with acetic acid and indigo carmine ( Fig. 1 **b** ), magnifying narrow-band imaging (M-NBI) was performed, which revealed disrupted glandular architecture and irregular microvasculature ( Fig. 1 **c** ). Due to the highly suspicious cancer-like appearance, we proceeded with confocal laser endomicroscopy (CLE), which showed overall preserved glandular structures with focal mild irregularities and polygonal epithelial changes, dark-stained goblet cells between epithelial cells in some glands, and extensive fluorescein leakage along with abundant microvasculature in the stroma ( Fig. 2 , Video 1 ). Based on CLE imaging, inflammatory changes with intestinal metaplasia and focal atypia were suspected. Subsequent histopathology confirmed mild to moderate chronic active gastritis with prominent plasma cell infiltration, without evidence of malignancy ( Fig. 3 ). CLE findings and serological results were combined, and a final diagnosis of gastric syphilis was established. The patient received standard benzathine penicillin therapy for three weeks with symptom relief. Follow-up endoscopy one month later showed ulcer healing ( Fig. 4 **a, b** ), and M-NBI demonstrated normalization of glandular and vascular patterns ( Fig. 4 **c** ).

**Fig. 1:**
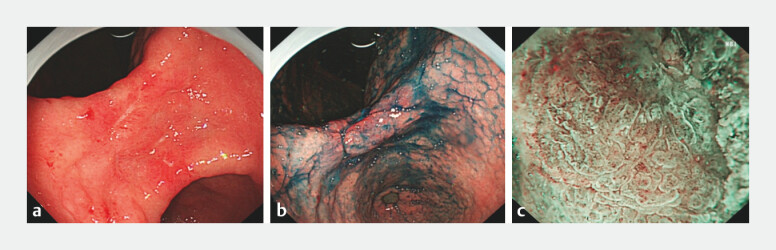
**a** Irregular ulcer at the gastric angle under white-light endoscopy. **b** Acetic acid-indigo carmine staining of the lesion. **c** Magnifying narrow-band imaging (M-NBI) shows disrupted glandular architecture and irregular microvasculature around the ulcer.

**Fig. 2:**
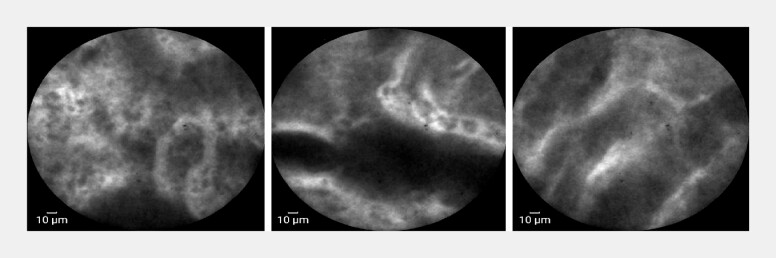
Confocal laser endomicroscopy reveals overall preserved glandular structures with mild focal irregularities. Dark-stained goblet cells visible between epithelial cells; extensive fluorescein leakage and abundant microvasculature in the stroma.

**Fig. 3:**
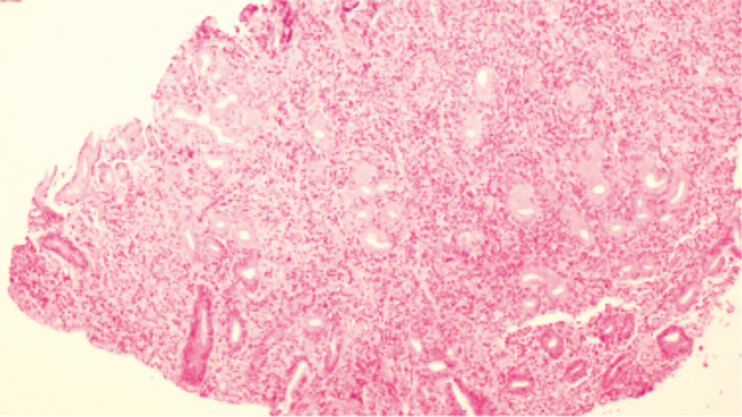
Hematoxylin and eosin staining (×40) shows marked plasma cell infiltration in the lamina propria and mild to moderate active inflammation; no cytological atypia observed.

**Fig. 4:**
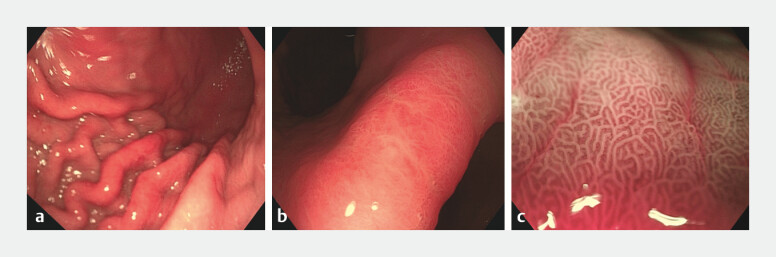
**a, b** White-light endoscopic images of the gastric body and angle after treatment. **c** M-NBI shows normalized glandular and vascular architecture at the gastric angle.

Confocal laser endomicroscopy of the gastric lesion showing preserved glandular architecture, dark goblet cells, and extensive fluorescein leakage in the stroma.Video 1

This case highlights the utility of CLE in accurately distinguishing benign lesions from cancer-mimicking findings when conventional endoscopy results are suspicious but histology is inconclusive. Approximately 17% of gastric syphilis cases have reportedly undergone unnecessary surgery due to misdiagnosis 1 . CLE enables real-time visualization of cellular and microvascular structures, providing critical information for differentiating ulcer etiology and malignancy 2 . With its “optical biopsy” capability, CLE can reduce reliance on conventional tissue biopsies and significantly improve diagnostic efficiency and accuracy, showcasing its promising potential in precision endoscopic diagnostics.

Endoscopy_UCTN_Code_CCL_1AB_2AC_3AB

## Notes

Endoscopy E-Videos https://eref.thieme.de/e-videos
*E-Videos* is an open access online section of the journal *Endoscopy* , reporting on interesting cases and new techniques in gastroenterological endoscopy. All papers include a high-quality video and are published with a Creative Commons CC-BY license. Endoscopy E-Videos qualify for HINARI discounts and waivers and eligibility is automatically checked during the submission process. We grant 100% waivers to articles whose corresponding authors are based in Group A countries and 50% waivers to those who are based in Group B countries as classified by Research4Life (see: https://www.research4life.org/access/eligibility/ ). This section has its own submission website at https://mc.manuscriptcentral.com/e-videos .
